# Osteopathic Manipulative Treatment Relieves Post-concussion Symptoms in a Case of Polytrauma

**DOI:** 10.7759/cureus.7317

**Published:** 2020-03-18

**Authors:** Gerard A Baltazar, Christine Kolwitz, Patrizio Petrone, Adam Stright, D'Andrea Joseph

**Affiliations:** 1 Surgery, NYU Langone Health/NYU Winthrop Hospital, Mineola, USA

**Keywords:** trauma, concussion, osteopathic manipulative treatment

## Abstract

Optimal management of post-concussion symptoms (PCS) remains ill-defined but includes multimodal, symptom-guided plans of care. Osteopathic manipulative treatment (OMT) may be used as an adjunct treatment for PCS.

We present a case of a motor vehicle collision victim whose PCS improved directly and progressively after OMT. To our knowledge, this is the first report of OMT utilized for PCS management after polytrauma and as part of an organized trauma system.

Previous studies discuss potential benefits of OMT for patients with PCS after sports-related injuries, and none account for management of multiply injured patients as part of an organized trauma system. Further study of OMT for PCS is warranted and would benefit by recruiting patients from trauma centers in order to observe a range of mechanisms of injury that result in concussion.

## Introduction

The Centers for Disease Control and Prevention estimates 3.2 to 5.3 million people in the United States live with concussion-related disability [1]. Optimal treatment for post-concussion symptoms (PCS) remains ill-defined but includes multimodal, symptom-guided plans of care [2]. 

Osteopathic manipulative treatment (OMT) is a set of hands-on techniques to diagnose and treat illness or injury. OMT generally involves movements of a patient’s muscles and joints using techniques that include stretching, gentle pressure and resistance [3]. OMT in the cranial field involves the gentle application of manual force primarily to the head that affects the primary respiratory mechanism and may provide benefits for pain, vision, autonomic regulation and sleep quality [4].

We present a multiply injured patient who suffered a motor vehicle collision (MVC) with soft tissue injury, skull and orthopedic fractures and persistent PCS. Her PCS improved directly and progressively after OMT sessions. To our knowledge, this is the first description of OMT utilized for PCS management after polytrauma and as part of an organized trauma system.

## Case presentation

**Figure 1:**
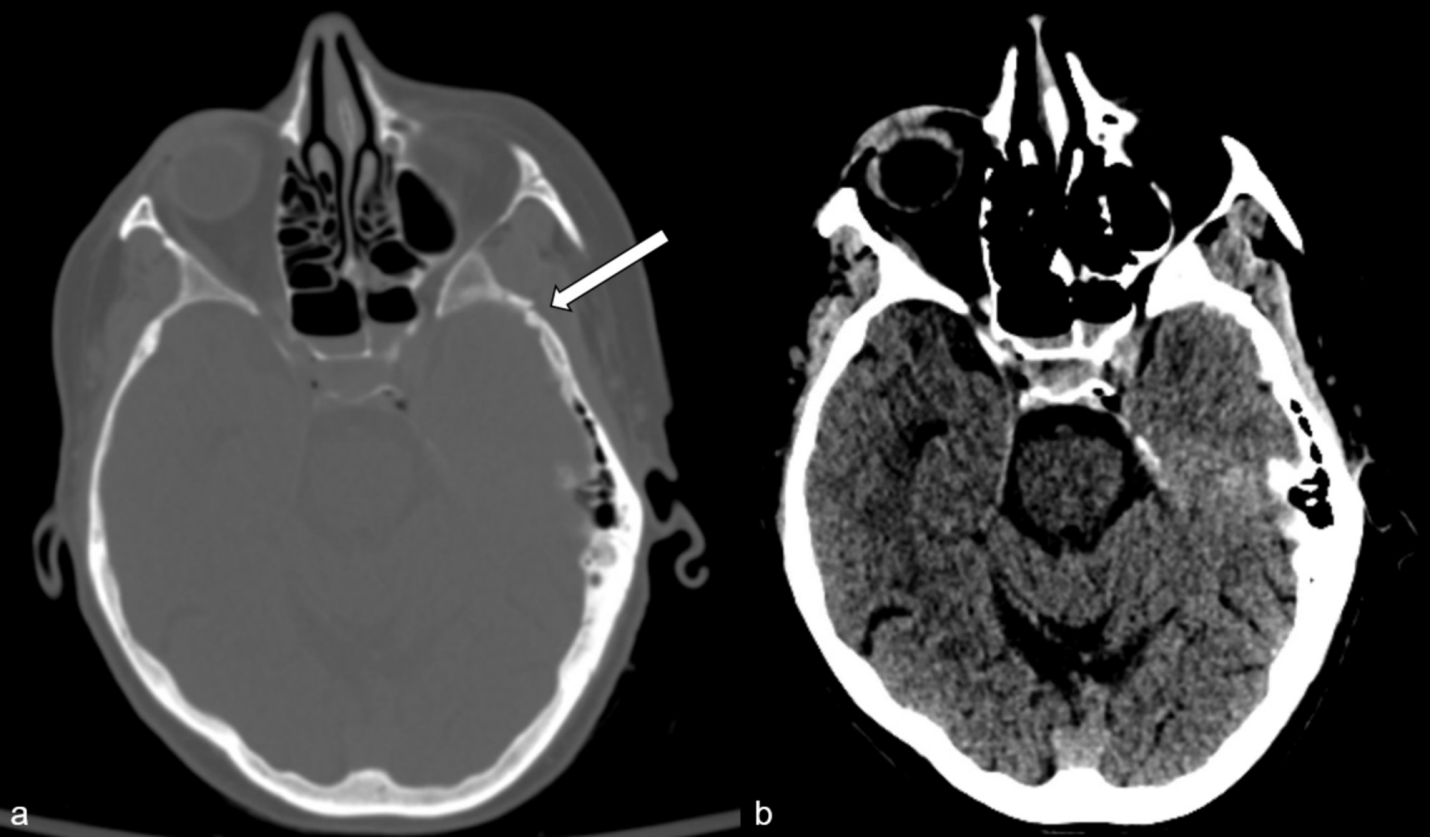
Computerized tomography demonstrates soft tissue contusion and skull fracture (a, arrow) but no intracranial hemorrhage (b).

A 66-year-old female with no significant past medical history presented as a trauma system activation after a high-speed MVC. Workup revealed left squamous temporal bone fracture with extension to the sphenoid sinus, associated intrasinus hemorrhage and minimal pneumocephalus, right frontal sinus fracture with extension into the right orbital roof, left frontal scalp hematoma, left supraorbital laceration and non-displaced left clavicle fracture. Computerized tomography did not reveal intracranial hemorrhage (Figure 1).

The laceration was repaired, and the clavicle fracture was treated with a sling. She was observed in hospital for two days for pain control and physical therapy, and then discharged to home in stable condition.

First follow-up in the trauma clinic on post-injury day 9 revealed left parietal, neck and shoulder pain, anhedonia, agitation and insomnia for which watchful waiting and analgesics were prescribed. She completed follow-up with orthopedics and neurosurgery, who also recommended watchful waiting and analgesics. 

Five weeks later, she presented to trauma clinic complaining of worsening headaches to the left occipital, temporal and parietal areas and persistent decreased activity level. Osteopathic cranial examination revealed sphenobasilar synchondrosis compression, left occipitomastoid compression, left temporal bone external rotation, bilateral frontal compression, decreased cranial rhythmic impulse and suboccipital muscle spasm. The patient was advised of possible benefits from and literature on OMT after head injury.

She returned to the office two weeks later for a planned OMT session. The headaches had become more intense and persistent, were associated with worsening left neck pain and interfered with concentration and sleep. OMT was performed and targeted cranial, cervical spine and bilateral upper extremity somatic dysfunctions. Gentle myofascial release, balanced ligamentous tension, paraspinal inhibition and OMT in the cranial field were performed over the course of 35 minutes with a focus on achieving sutural decompression and alignment, restoration of intracranial fluid motion and increase of locoregional vertebral and soft tissue mobility. This session resulted in immediate and progressive improvement of neck, head and left upper extremity pain and mobility. The head and neck pain ceased completely one day later, the first time she had been pain-free since the MVC. Her sleep quality significantly improved, allowing increased activity level. Her self-reported use of analgesics decreased. 

She returned one month later for planned OMT. The intense head and neck pain had not recurred. However, she reported left visual field central and lateral gaze blurriness since the MVC for which she had seen a neuro-ophthalmologist in the interim, who advised watchful waiting. This symptom had become more apparent and bothersome since relief of the previous pain syndrome. She also noted left periauricular paresthesia, mild dizziness and mild left temple pain localized to the intersection of the frontal and sphenoid sutures which had also become more apparent. 

Gentle OMT focused on the treatment of left sphenoid torsion, left rib exhalation dysfunction, diaphragmatic motion and soft tissue somatic dysfunction. She experienced immediate improvement of pain and periauricular paresthesia and transient improvement of dizziness and blurriness. Thereafter, she was able to return to driving in a limited fashion.

She returned one month later complaining of improved but persistent left temple pain and visual field blurriness. An extended, 45-minute treatment with OMT in the cranial field resulted in immediate and progressive improvement in pain, dizziness and visual field blurriness. Thereafter, she was able to return to full athletic activity and had increased driving ability.

Subsequent sessions of OMT consisted of reassessment and treatment of full-body somatic dysfunction and optimization of cranial function, including use of Fulford’s dural release performed as follows: the patient is asked to gaze in a right or left direction, and the physician rotates the head in the same direction up to the restrictive end point of rotation; the patient then gazes in the other direction as the physician rotates the head to the other end point of rotation; this is subsequently performed with rotation and gaze in opposing directions; each maneuver is repeated until fluid motion is achieved [5]. She experienced immediate and continued improvement of her visual field after each session.

Throughout her course, no aggressive OMT techniques (e.g. high-velocity, low-amplitude “popping” technique) and no additional complementary and alternative medicine modalities were used, and no major manual therapy adverse events(e.g. neurovascular injury) occurred [6]. No quantitative PCS test was used to monitor the progress of this patient.

## Discussion

OMT may be a beneficial adjunct for the care of concussed patients [5,7-10]. Three case reports describe patients with isolated head injuries whose PCS improved after outpatient OMT [5,7,8].^ ^ A prospective case series describes an association between a focused, five-day craniosacral manipulation regimen and immediate and long-term PCS improvement [9]. A retrospective review demonstrated immediate improvements in the Standardized Concussion Assessment Tool 2 after one OMT session [10]. All the aforementioned cases describe sports-related injuries treated in outpatient settings. We present the first case of PCS after non-sports-related polytrauma treated with OMT. With watchful waiting and analgesia, the patient demonstrated no improvement and only had alleviation of PCS after implementation of OMT.

Our patient’s outcome is most similar to reports by Yao and Castillo et al., who describe profound improvement of PCS after an initial session of OMT followed by progressive resolution of PCS, including significant progress immediately after subsequent OMT sessions [5,7].

The optimal regimen of OMT for PCS is not defined and will likely be patient and injury specific, particularly if the patient has suffered polytrauma. In our case, frequency of OMT sessions was monthly due to resource limitations (i.e. only one trauma surgeon at our institution provides OMT [GB]), and six sessions were required for return to near-full activity. In contrast, Castillo et al. provided six weekly OMT sessions and Yaodescribed five biweekly OMT sessions before return to functional activity level [5,7]. Based on more rapid benefits in these similar cases, more frequent opportunities for OMT post-trauma activation for concussion seem warranted.

Similarly, optimal timing to start and frequency of OMT sessions for PCS have not been established as national osteopathic organizations do not provide guidance on the subject. OMT was first performed for our patient approximately six weeks post-injury in part to allow completion of other subspecialist follow-up, and fracture healing-acute fracture is a relative contraindication to OMT of the fracture [11]. In contrast to our case, Guernsey et al. achieved complete resolution of PCS after acute isolated head injury (three days prior to contact) through a single OMT session, suggesting that earlier OMT may be more effective at treating PCS [8]. 

Chronicity of PCS also does not seem to prohibit improvement after OMT, as Wetzler et al. describe former National Football League players who suffered years of head injuries experiencing significant improvement after five days (10 sessions) of focused craniosacral manipulation [9]. Wetzler et al. also describe follow-up with improvement in PCS three months after OMT, suggesting a potential for long-term positive outcomes for PCS after OMT [9].

The underlying pathophysiology of PCS is not well defined nor is the cause of benefit of OMT for PCS [2]. Zwibel et al. provide a summative outline of an osteopathic approach to concussion, including various quantitative assessment tools for patients with concussion, osteopathic structural examination and patient-specific OMT [12]. Several authors describe underlying physiological benefits of OMT for symptoms associated with concussion, including dizziness and imbalance, tension-type headache, autonomic dysregulation and modulation of the glympathic system [7,13-18].

## Conclusions

Given limited targeted treatment regimens for PCS and the reported profound benefits of OMT, further utilization and study of OMT for PCS are warranted. Future work should use a quantitative assessment tool to track patient progress and would benefit by recruiting patients from trauma centers in order to observe a range of mechanisms of injury that result in concussion.
